# Factors Associated With Turnover Intention Among Healthcare Providers in Saudi Arabia: A Comprehensive Review

**DOI:** 10.7759/cureus.69565

**Published:** 2024-09-16

**Authors:** Khalid Alkhurayji, Asma Alshehri, Nuwayyir Albalawi, Maram Alshahrani, Nouf Alotaibi, Najla Assiri, Turki Alhabib, Deena Alghouth, Nouf Alqahtani, Bandar Aldosari

**Affiliations:** 1 Dental Center, Prince Sultan Military Medical City, Riyadh, SAU; 2 Dental Services, Ministry of National Guard Health Affairs, Riyadh, SAU; 3 Emergency Department, Prince Sultan Military Medical City, Riyadh, SAU

**Keywords:** healthcare, healthcare provider, hospitals, sustainability, turnover

## Abstract

Turnover has a negative impact and can harm businesses, specifically the organizational structure and service delivery. The purpose of this review is to provide policymakers, healthcare administrators, and stakeholders in Saudi Arabia with approaches and strategies for reducing turnover and improving provider retention by synthesizing existing literature and identifying the key variables influencing turnover intention.

This study conducted a comprehensive literature review to explore numerous variables associated with medical professional turnover intentions in Saudi Arabia from 2016 to 2024. The librarian assisted with the creation of the query string. The literature search was carried out using PubMed, Web of Science, and Google Scholar. By utilizing the key terms turnover AND healthcare providers AND healthcare facilities AND Saudi*. The authors separately examined and evaluated the risk of bias in each study using Joanna Briggs Institute (JBI) tools.

The findings of this review show that all studies were executed using a cross-sectional design, and five studies were published in 2022. According to this review, ten studies were carried out in hospital settings. Ten studies examined elements associated with work. Moreover, five studies were associated with external variables, followed by five studies linked to personal factors. The most common factors for turnover were satisfaction, stress, and work.

Turnover-related factors can have an impact on the long-term sustainability of the healthcare system. This review provides crucial information on the factors related to turnover, using the growing body of research in the Saudi context.

## Introduction and background

Turnover has negative implications and can harm enterprises, specifically the organization’s structure and service delivery [1-3]. Previous studies demonstrated that turnover not only harms the organization financially through selection or recruitment processes and termination charges, but it can also have a negative impact on other staff members completing their assigned jobs [4].

Several organizations lose service provision, particularly when an employee has exceptional performance, which exemplifies the negative effects of organizational sustainability. As a result, the difficulty of recruiting, training, and replacing departing employees affects the entire system [5,6].

In healthcare organizations, provider turnover remains a major challenge, particularly in Saudi Arabia [7]. High turnover rates among healthcare providers can negatively affect patient care quality, the effectiveness of organizations, and overall system performance [8]. Therefore, understanding the factors influencing health professionals' intention to leave is crucial for developing effective retention strategies and improving workforce stability.

The current study looks at the factors that influence healthcare professionals' intentions to leave organizations in Saudi Arabia. The goal of this study was to identify, evaluate, and synthesize all studies on turnover-related variables to assist policymakers, healthcare administrators, and stakeholders in Saudi Arabia with approaches and strategies for minimizing turnover and improving provider retention.

## Review

Methodology

This study conducts a comprehensive literature analysis to identify factors influencing healthcare professional turnover intentions in Saudi Arabia from 2016 to 2024. This research included a thorough evaluation and synthesis of existing studies undertaken in the Saudi healthcare industry to identify common themes and major drivers influencing turnover intention among healthcare providers using the Turnover Humanitarian Organizations Framework [9]. This study concentrated on healthcare providers in the Kingdom of Saudi Arabia, including hospitals and primary, secondary, and tertiary care centers in various parts of the country. By focusing on Saudi Arabia, the study attempted to provide insights into the distinct organizational, cultural, and societal elements that contribute to turnover intention among healthcare providers.

The review only covered studies on healthcare practitioners. The main outcome of the review was the turnover factors associated with healthcare providers. Additionally, the study must be conducted in a hospital or other healthcare facility to be included in this evaluation. We omitted studies in which healthcare practitioners did not offer medical care.

The librarian assisted in constructing the search string. This search string incorporated filters, synonyms, mesh, and other subject terms by utilizing the key terms turnover AND healthcare providers AND healthcare facilities AND Saudi* (see Appendix 1). PubMed, Web of Science, and Google Scholar were used for literature searches. We concentrated mainly on publications of peer-reviewed research articles. We also evaluated the reference lists of the included papers. However, we excluded theses, conference abstracts, in-press articles, and book chapters. In addition, we limited our search to English-language content.

Following the title and abstract screening, the authors obtained the full text of the remaining articles and compared the whole text to the inclusion criteria. After acquiring and reviewing the entire text, the authors performed a citation search. Discrepancies were resolved through consensus.

The authors extracted the data along with each study's features and outcomes using a standardized data extraction form that was initially tested on five studies. The authors used JBI tools to examine and assess the risk of bias in each study separately [10]. Where data was missing, the research authors or sponsors were contacted.

Result

Depicts the search strategy employed in this research, resulting in the identification of 763 records from three databases. After the 220 duplicate records were removed. The title and abstract were scanned, which eliminated 177 records, resulting in the inclusion of 366 studies. These papers were then assessed, and 306 were removed based on inclusion and exclusion criteria. During the retrieval phase, 13 of the 60 studies were not retrievable. Following the evaluation of eligibility for the full text of 47 studies, 20 studies were removed for lack of evidence and 13 for measuring outcomes not related to turnover, which resulted in the inclusion of 14 studies.

This review revealed that 5 of the 14 papers were published in 2022. In addition, two studies were released in 2018, followed by four investigations in 2021. There was, however, only one study published in 2023. Additionally, a study was conducted in 2024. Furthermore, all studies used a cross-sectional design, and 8 of the 14 were done in hospitals. However, one study took place in a tertiary hospital, another in a secondary hospital, and one online.

Table 1 demonstrates that four studies were undertaken with healthcare professionals. However, eight studies were conducted among nurses. Moreover, the work-related dimension contained 10 studies that were linked to turnover intentions. Additionally, five studies were associated with external factors. However, personal factors were detected in five of the studies included in this review. furthermore, The JBI tool checklist contains eight questions to determine the risk of bias for cross-sectional design studies. Most investigations produce excellent results, according to the risk of bias evaluation (Appendix 2).

**Table 1:** Studies characteristic, factors, dimensions, and assessment of bias RoB (risk of bias): Values of 50% or below indicate a low-quality article, between 50% and 69% are considered average quality, and 70% represent the high quality of the article.

study	Year	research design	Context	population	factors of turnover	Dimension	Summary	Risk of Bias (RoB)
Albazroun [8]	2023	Cross-sectional	Hospital	Nurse	Age, sex, nationality, salary, years of experience, and shift length	Work-related factors, personal factor	There was a significant inverse relationship between job satisfaction and intention to leave.	100%
Almuharraq [11]	2022	Cross-sectional	Tertiary medical hospital	Nurse	Workplace bullying	Work-related factors	There was a positive significant correlation between workplace bullying and turnover intentions.	87.5%
Almansour [12]	2021	Cross-sectional	Primary healthcare center	Healthcare workers	Stress, social support	Personal factors, external factors	Stress is associated with turnover intention among healthcare workers in Saudi Arabia. Social support had a mitigating effect on the relationship between stress and turnover intention.	100%
Alanezi [13]	2022	Cross-sectional	Hospital	Healthcare workers	Compensation, workload, possibilities for career growth, and the atmosphere of the workplace	Work-related factors	Current nursing shortage and high turnover because of the effects they have on the efficacy and efficiency of any system that delivers healthcare.	75%
Alblihed [14]	2022	Cross-sectional	Hospitals	Health workers	Job stress, role ambiguity, and work-life Imbalance	External factors, work-related factors	The findings show that job burnout is clearly related to turnover intentions and is positively affected by both role stress and role ambiguity.	87.5%
Albougami [15]	2020	Cross-sectional	Hospitals	Nurse	Quality of life, stress, and job satisfaction	Work-related factors, external factors	Quality of life dimensions, such as physical and psychological health, predict nurses’ intention to resign from their current workplaces.	87.5%
Alharthi [16]	2024	Cross-sectional	Hospitals	Nurse	Professional personal goals at work, family's opinion about the nursing profession, work interferes with family responsibilities, work interferes with family responsibilities, and impact of family life on the professional duties	Personal factors, work-related factors	There was a direct significant statistical relation between some demographic characteristics (age, marital status, nurse guardian, current job position, having kids, duration in the nursing profession), and nurse turnover or intention to leave the profession.	87.5%
Aljohani [17]	2018	Cross-sectional	Hospitals	nurse	Financial, administration support, quality of life, and work environment	Work-related factors, external factors, personal factors	The top cause of turnover was low salaries, despite the nurses being aware of their salary before signing the contract.	100%
Almohammadi [18]	2022	Cross-sectional	Hospitals	Nurse	Family obligations, guardian's decision, society's perception	external factor	The reality of turnover, which is impacted by variables such as low compensation, restricted career growth prospects, hard workloads, and burnout, causes disruptions in the stability of healthcare teams.	87.5%
[19] Alotheimin	2023	Cross-sectional	Hospital	Nurse	Happiness	Personal factor	The relationship between happiness and turnover intentions is negative.	87.5%
Alreshidi [20]	2021	Cross-sectional	Hospital	Nurse	Professional growth and development, leadership style, management, wage and benefits, workload, interpersonal relationship, housing facilities and services, and hospital facilities	Work-related factors	Wage benefits and workload factors were found to be the most significant causes of expatriate nursing turnover, closely followed by inadequate housing and hospital facilities.	87.5%
Attar [21]	2021	Cross-sectional	Hospital	Nurse	Organizational culture, job stress and workload, salary, and beneficiary factors	Work-related factors	The workload is statistically significant, with nurses' turnover. The organizational culture is the strongest contributing factor while the workload factors are shown to be the least strong contributing factor to why nurses are leaving their jobs.	87.5%
Kaddourah [22]	2018	Cross-sectional survey	Tertiary medical hospital	Nurse	Quality of nursing work life	Work-related factors	There is a low indication of satisfaction of nurses with their quality of nursing work life and a high turnover intention.	87.5%
Nassani [23]	2021	Cross-sectional survey	Online-based	Healthcare workers	Work stress, job satisfaction	Work-related factors	There is a significant positive effect between work stress and turnover intention, and a significant negative effect between job satisfaction and turnover intentions.	75%

Work-related factors involved in turnover intention demonstrate that work interferes with family life and may influence the intention to leave, in addition to salary, years of experience, and shift length [8,16]. Workplace bullying negatively impacts employees and increases their desire to leave. Similarly, workload and working conditions show an unfavorable influence on turnover intention [11,13]. On the other hand, opportunities for career progression, development, salary, and leadership may assist lower turnover intentions [14,20,21]. Other aspects include work in the organization such as support and organizational culture [15-22].

The personal factors associated with turnover indicate that personal goals, satisfaction, and age may increase the likelihood of turnover among healthcare workers, particularly nurses [8,12,16,17,19]. This review identifies extrinsic influences such as social support, quality of life, family obligations, guardianship decisions, and societal perception [12,14,15,17,18].

**Figure 1:**
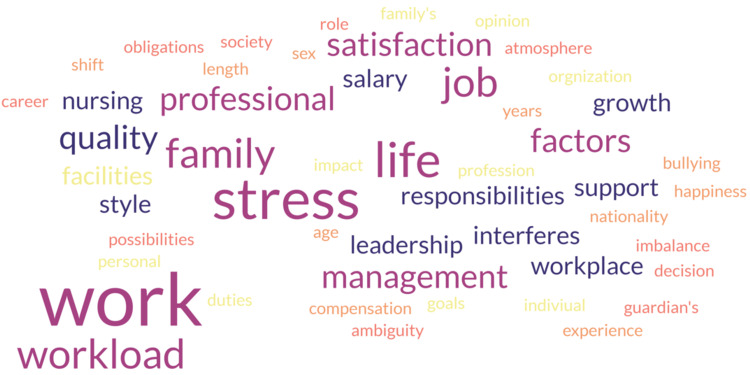
Word cloud of the factors associated with turnover The figure is the author's creation. The word cloud represents factors associated with turnover intention. Each word's size reflects its frequency.

Figure 1 depicts the factors associated with turnover, among which the most common factors in this review are satisfaction, stress, and work.

Discussion

Based on our thorough analysis, the majority of reasons influencing turnover are work-related. Nonetheless, personal and environmental factors influenced turnover in both positive and negative directions. However, workload and stress were prevalent themes in this review. The previous review highlighted factors in nursing specialties that influence turnover intention such as organizational structure, workload, stress, management style, and individual factors [24]. Nonetheless, this review found factors implicated in the personal, external, and work-related domains.

Albalawi and Pascua discovered that individual, occupational, and organizational factors all contributed to turnover [25]. However, Falatah and Salem found that demographics, enjoyment, management, and job-related factors were all associated with turnover among nurses [26]. Similarly, this study discovered that learning, job, and satisfaction all contributed to turnover.

Work and personal factors, such as age, salary, years of experience, and shift length, were prevalent among nursing specialties working in hospitals [8]. However, those who worked in tertiary hospitals demonstrated that workplace bullying was prevalent in these settings [11]. According to Kaddourah [22] and Albougami [15], nurses in tertiary medical centers and hospitals have low job satisfaction due to quality of life, stress, and job satisfaction. The variability of these characteristics could be attributable to numerous factors related to the types of health institutions, job conditions, and patients' volume.

Another point of view, according to Alharthi and Aljohani, nursing professionals in hospitals are influenced by more than only personal and work-related factors [16,17]. Hence, external factors may have driven them to leave the institute given to interference with family obligations and the impact of family life on their overall quality of life. Similarly, Almohammadi specified that family duties, Guradin's decision, and society all have an impact on the intention to leave the health organization [18]. However, Alotheimin found that happiness can reduce turnover intuition [19]. Overall, nursing in Saudi Arabia, in different contexts, can handle the workload [21]. However, without specific professional growth and development, a lack of excellent organizational culture, and beneficial aspects, turnover may increase significantly [20].

Healthcare workers are faced with several reasons that influence their decision to leave organizations, including personal factors, such as stress, and external factors such as social support [12]. However, Alanezi observed that health workers in Saudi Arabia have high turnover due to factors such as compensation, workload, and work-related factors [13]. Albilhed stated that healthcare personnel encounter external and work-related factors that contribute to job turnover and work-life balance issues [14]. Despite this, Nassani believes that organizations that do not focus on enhancing work satisfaction will increase employee turnover [23]. These aspects can have different effects due to the large variation of job types and the context of hospitals and services provided among healthcare providers.

Using several databases and search engines, this study investigated the factors that contribute to high turnover among healthcare workers. The study examined three dimensions: personal, external, and work-related. Nonetheless, to our knowledge, no previous study has evaluated and summarized the full body of knowledge on the reasons of turnover in Saudi Arabia. Furthermore, this study may assist decision-makers in reducing turnover and improving retention methods by identifying traits common among healthcare practitioners. However, the study had certain limitations. First, adding more databases to search engines can increase the accuracy of the results. Second, due to English language restriction, some research may have been missed. Third, combining multiple risk assessment approaches may improve the critical evaluation of the research in this review.

Despite these limitations, this study addresses knowledge gaps, particularly in the Saudi context. Research in these areas has the potential to accelerate strategic progress towards lowering turnover rates. Furthermore, future research should discover additional factors that influence turnover intention and perform ongoing assessments in various geographical regions to improve the generalizability of the findings worldwide.

In the context of Saudi Arabia, the current directions are based on nursing specialists' turnover intentions. Nonetheless, multidisciplinary healthcare workers face numerous elements that contribute to turnover intentions. Future direction should focus on identifying more specific challenges faced by healthcare workers to address them and boost the resiliency of the health system and healthcare services. The recommendations of this comprehensive review are to make additional initiatives to improve retention among healthcare workers, primarily nursing specialists. The factors identified in this review in terms of population, context, and domains can regulate the intervention of policymakers and leaders of health institutions to increase the satisfaction of overall health workers.

## Conclusions

This review provides critical details on the factors associated with turnover that integrate the growing body of knowledge in the Saudi Arabian context. Factors associated with turnover can impact the sustainability of the healthcare system, particularly in hospital settings. Managers, decision-makers, and policy designers must implement strategies to overcome these factors to improve the efficiency and effectiveness of the healthcare system.

## Appendices

Appendix 1: search strategies

PubMed - run 10/6/2024 (turn-over)) OR (turnover)) AND (healthcare providers[MeSH Terms])) OR (medical staff[MeSH Terms])) AND (hospitals[MeSH Terms])) OR (primary care[MeSH Terms])) OR (secondary care[MeSH Terms])) OR (tertiary care[MeSH Terms])) OR (healthcare facilities) ) AND (Saudi Arabia*)) OR (saudi*))

Web of Science - run 10/6/2024 TS=("turn-over" OR "turnover") AND TS=("healthcare providers" OR "medical staff") AND TS=("hospitals" OR "primary care" OR "secondary care" OR "tertiary care" OR "healthcare facilities") AND (“Saudi Arabia*) OR (Saudi*)

Google Scholar - run 10/6/2024 ("turn-over" OR "turnover") AND ("healthcare providers" OR "medical staff") AND ("hospitals" OR "primary care" OR "secondary care" OR "tertiary care" OR "healthcare facilities") AND (“Saudi Arabia*) OR (Saudi*)

Appendix 2: risk of bias assessment 

**Table 2:** Risk of bias assessment of the JBI tool Values of 50% or below indicate a low-quality article; between 50% and 69% are considered average quality; and 70% represent the high quality of the article. (Yes “1”, No “0”, undefined “UND”, and not applicable “-”) JBI: Joanna Briggs Institute

Reference	1	2	3	4	5	6	7	8	Total	%
[16]	1	1	1	1	1	0	1	1	7 (8)	87.5
[8]	1	1	1	1	1	1	1	1	8 (8)	100
[19]	1	1	1	1	1	0	1	1	7 (8)	87.5
[11]	1	1	0	1	1	1	1	1	7 (8)	87.5
[13]	1	1	0	1	1	0	1	1	6 (8)	75
[14]	1	1	1	1	1	0	1	1	7 (8)	87.5
[18]	1	1	1	1	1	0	1	1	7 (8)	87.5
[12]	1	1	1	1	1	1	1	1	8 (8)	100
[20]	1	1	1	1	0	1	1	1	7 (8)	87.5
[21]	1	1	1	1	1	0	1	1	7 (8)	87.5
[23]	1	1	0	1	1	0	1	1	6 (8)	75
[15]	1	1	1	1	1	0	1	1	7 (8)	87.5
[17]	1	1	1	1	1	1	1	1	8 (8)	100
[22]	1	1	1	1	1	0	1	1	7 (8)	87.5
